# Fast machine learning annotation in the medical domain: a semi-automated video annotation tool for gastroenterologists

**DOI:** 10.1186/s12938-022-01001-x

**Published:** 2022-05-25

**Authors:** Adrian Krenzer, Kevin Makowski, Amar Hekalo, Daniel Fitting, Joel Troya, Wolfram G. Zoller, Alexander Hann, Frank Puppe

**Affiliations:** 1 Department of Artificial Intelligence and Knowledge Systems, Sanderring 2, 97070 Würzburg, Germany; 2 Interventional and Experimental Endoscopy (InExEn), Department of Internal Medicine II, University Hospital Würzburg, Oberdürrbacher Straße 6, 97080 Würzburg, Germany; 3 Department of Internal Medicine and Gastroenterology, Katharinenhospital, Kriegsbergstrasse 60, 70174 Stuttgart, Germany

**Keywords:** Machine learning, Deep learning, Annotation, Endoscopy, Gastroenterology, Automation, Object detection

## Abstract

**Background:**

Machine learning, especially deep learning, is becoming more and more relevant in research and development in the medical domain. For all the supervised deep learning applications, data is the most critical factor in securing successful implementation and sustaining the progress of the machine learning model. Especially gastroenterological data, which often involves endoscopic videos, are cumbersome to annotate. Domain experts are needed to interpret and annotate the videos. To support those domain experts, we generated a framework. With this framework, instead of annotating every frame in the video sequence, experts are just performing key annotations at the beginning and the end of sequences with pathologies, e.g., visible polyps. Subsequently, non-expert annotators supported by machine learning add the missing annotations for the frames in-between.

**Methods:**

In our framework, an expert reviews the video and annotates a few video frames to verify the object’s annotations for the non-expert. In a second step, a non-expert has visual confirmation of the given object and can annotate all following and preceding frames with AI assistance. After the expert has finished, relevant frames will be selected and passed on to an AI model. This information allows the AI model to detect and mark the desired object on all following and preceding frames with an annotation. Therefore, the non-expert can adjust and modify the AI predictions and export the results, which can then be used to train the AI model.

**Results:**

Using this framework, we were able to reduce workload of domain experts on average by a factor of 20 on our data. This is primarily due to the structure of the framework, which is designed to minimize the workload of the domain expert. Pairing this framework with a state-of-the-art semi-automated AI model enhances the annotation speed further. Through a prospective study with 10 participants, we show that semi-automated annotation using our tool doubles the annotation speed of non-expert annotators compared to a well-known state-of-the-art annotation tool.

**Conclusion:**

In summary, we introduce a framework for fast expert annotation for gastroenterologists, which reduces the workload of the domain expert considerably while maintaining a very high annotation quality. The framework incorporates a semi-automated annotation system utilizing trained object detection models. The software and framework are open-source.

## Background

Machine learning especially deep learning is becoming more and more relevant in research and development in the medical domain [1, 2]. For all of the supervised deep learning applications, data is the most critical factor in securing successful implementation and sustaining progress. Numerous studies have shown that access to data and data quality are crucial to enable successful machine learning of medical diagnosis, providing real assistance to physicians [3–7]. Exceptionally high-quality annotated data can improve deep learning detection results to great extent [8–10]. E.g., Webb et al. show that higher data quality improves detection results more than using larger amounts of lower quality data [11]. This is especially important to keep in mind while operating in the medical domain, as mistakes may have fatal consequences.

Nevertheless, acquiring such data is very costly particularly if domain experts are involved. On the one hand domain, experts have minimal time resources for data annotation, while on the other hand, data annotation is a highly time-consuming process. The best way to tackle this problem is by reducing the annotation time spend by the actual domain expert as much as possible while using non-experts to finish the process. Therefore, in this paper, we designed a framework that utilizes a two-step process involving a small expert annotation part and a large non-expert annotation part. This shifts most of the workload from the expert to a non-expert while still maintaining proficient high-quality data. Both of the tasks are combined with AI to enhance the annotation process efficiency further. To handle the entirety of this annotation process, we introduce the software Fast Colonoscopy Annotation Tool (FastCat). This tool assists in the annotation process in endoscopic videos but can easily be extended to any other medical domain. In the domain of endoscopic imaging, the main issue of clinical experts is to find and characterize pathologies, e.g., polyps in a screening colonoscopy. Thereby, the endoscopist examines the large intestine (colon) with a long flexible tube that is inserted into the rectum. A small camera is mounted at the end of the tube, enabling the physician to look inside the colon. The images from this camera can be captured and annotated to enable automatic real-time detection and characterization of pathologies [12, 13]. This process and other applications all need annotated data to enable high-quality results.

The main contributions of our paper are:

(1): *We introduce a framework for fast expert annotation, which reduces the workload of the domain expert while maintaining very high annotation quality.*
(2): *We publish an open-source software for annotation in the gastroenterological domain and beyond, including two views, one for expert annotation and one for non-expert annotation*.^1^
(3): *We incorporate a semi-automated annotation process in the software, which reduces the annotation time of the annotators and further enhances the annotation process’s quality. *

To overview existing work and properly allocate our paper in the literature, we describe a brief history reaching from general annotation tools for images and videos to annotation specialized for medical use.

### A brief history of annotation tools

As early as the 1990s, the first methods were conceived to collect large datasets of labeled images [14]. E.g., “The Open Mind Initiative”, a web-based framework, was developed in 1999. Its goal was to collect annotated data by web users to be utilized by intelligent algorithms [15]. Over the years, various ways to obtain annotated data have been developed. E.g., an online game called ESP was developed to generate labeled images. Here, two random online players are given the same image and, without communication, must guess the thoughts of the other player about the image and provide a common term for the target image as quickly as possible [14, 16]. As a result, several million images have been collected. The first and foremost classic annotation tool called labelme was developed in 2007 and is still one of the most popular open-source online annotation tools to create datasets for computer vision. Labelme provides the ability to label objects in an image by specific shapes, as well as other features [17]. From 2012 to today, with the rise of deep learning in computer vision, the number of annotation tools expanded rapidly. One of the most known and contributing annotation tools is LabelImg, published in 2015. LabelImg is an image annotation tool based on Python which utilizes bounding boxes to annotate images. The annotations are stored in XML files that are saved in either PASCAL VOC or YOLO format. Additionally, in 2015 Playment was introduced. Playment is an annotation platform to create training datasets for computer vision. It offers labeling for images and videos using different 2D or 3D boxes, polygons, points, or semantic segmentation. Besides, automatic labeling is provided for support. In 2017, Rectlabel entered the field. RectLabel is a paid labeling tool that is only available on macOS. It allows the usual annotation options like bounding boxes as well as automatic labeling of images. It also supports the PASCAL VOC XML format and exports the annotations to different formats (e.g., YOLO or COCO JSON). Next, Labelbox, a commercial training data platform for machine learning, was introduced. Among other things, it offers an annotation tool for images, videos, texts, or audios and data management of the labeled data.

Nowadays, a variety of image and video annotation tools can be found. Some have basic functionalities, and others are designed for particular tasks. We picked five freely available state-of-the-art annotation tools and compared them more in-depth. In Table 1, we shortly describe these tools and compare them.

**Table 1:** Comparison between video and image annotation tools

	Tool	CVAT	LabelImg	labelme	VoTT	VIA
	Image	$\bullet$	$\bullet$	$\bullet$	$\bullet$	$\bullet$
	Video	$\bullet$	-	-	$\bullet$	$\bullet$
	Usability	Easy	Easy	Medium	Medium	Hard
Formats	VOC	$\bullet$	$\bullet$	$\bullet$	$\bullet$	-
	COCO	$\bullet$	-	$\bullet$	-	$\bullet$
	YOLO	$\bullet$	$\bullet$	-	-	-
	TFRecord	$\bullet$	-	-	$\bullet$	-
	Others	-	-	$\bullet$	$\bullet$	$\bullet$

#### Computer Vision Annotation Tool (CVAT)

CVAT [18] was developed by Intel and is a free and open-source annotation tool for images and videos. It is based on a client-server model, where images and videos are organized as tasks and can be split up between users to enable a collaborative working process. Files can be inserted onto the server through a remote source, mounted file system, or uploading from the local computer. Before a video can be annotated, it must be partitioned into its frames, which then can be annotated. Several annotation formats are supported, including the most common formats such as VOC, COCO, YOLO and TFRecord. Available annotation shapes and types are labeling, bounding boxes, polygons, polylines, dots, and cuboids. CVAT also includes features for a faster annotation process in videos. The disadvantages of this tool are that it currently only supports the Google Chrome browser, and due to the Chrome Sandbox, performance issues could appear.

#### LabelImg

LabelImg [19] is an image annotation tool that is written in Python and uses the Qt framework as a graphical user interface. It can load a bulk of images but only supports bounding box annotations and saves it as a XML file in VOC or YOLO format. The functionalities are minimal but sufficient for manual annotation of images. Furthermore, it does not contain any automatic or semi-automatic features which could speed up the process.

#### labelme

The annotation tool labelme [20] is written in Python, uses Qt as its graphical interface and only supports image annotation. It is advertised that videos could be annotated with this tool, but no video annotation function was found and the user must manually extract all frames from the video beforehand. Also, there are no automatic or semi-automatic features available and uses basic shapes like polygons, rectangles, circles, points, lines and polylines to annotate images.

#### Visual Object Tagging Tool (VoTT)

Microsoft’s tool VoTT [21] is open-source and can be used for images and videos. Since it is written in TypeScript and uses the React framework as a user interface, it is possible to use it as a web application that can run in any web browser. Alternatively, it can also run locally as a native application with access to the local file system. Images and videos are introduced to the program via a connected entity. This can be a path on the local file system, a *Bing* image search query via an API key, or secure access to an *Azure Blob Storage* resource. Available annotation shapes are rectangles and polygons that can be tagged. These can then be exported for the *Azure Custom Vision Service* and *Microsoft Cognitive Toolkit* (CNTK).

#### VGG Image Annotator (VIA)

VIA [22, 23] is a tool that runs in a web browser without further installation and is only build from HTML, JavaScript, and CSS. It can import and export annotations from COCO and a VIA-specific CSV and JSON. The available annotation shapes are polygons, rectangles, ellipses, lines, polylines, and points. Video annotation features the annotation of temporal segments to mark, e.g., a particular activity within the video. Defined segments of the track can also annotate an audio file. VIA does not contain any automatic functionalities within the tool itself; these are relatively independent steps. These steps can be broken down to: Model predicts on frames, save predictions so that they can be imported into VIA, and lastly, check and update annotations if necessary.

### Medical annotation tools

With the considerable increase in interest and progress in machine learning in our society the need for machine learning models shifts in different domains including medicine. Thus, artificial intelligence can be used to assist medical professionals in their daily routines [24–26]. As a result, the need for labeled medical images and videos is also a major issue for medical professionals. While it is possible to use common annotation tools such as those already described above, some annotation tools have already been adapted to medical conditions. A well-known example from 2004 is “ITK-Snap”, a software for navigating and segmenting three-dimensional medical image data [27].

Another example is an open-source tool widely used in the medical domain called 3D slicer [28]. 3D slicer is a desktop software to solve advanced image computing challenges in the domain of medical applications. Thereby, it is possible to visualize special medical formats like DICOM (Digital Imaging and Communications in Medicine) in the tool and edit it with the 3D slicer software. Additionally, 3D Slicer incorporates Artificial Intelligence (AI) via AI-assisted segmentation extension in the 3D slicer software (DeepInfer, TOMAAT, SlicerCIP, Nvidia Clara). Thereby, automatic segmentations can be created and edited for, e.g., CT scans of brains.

“ePAD” is an open-source platform for segmentation of 2D and 3D radiological images [29]. The range of medical segmentation tools has become very broad nowadays, as they are usually specialized for many different areas of medicine.

Another annotation tool published in 2015 is TrainingData [30, 31]. TrainingData is a typical annotation tool for labeling AI (computer vision) training images and videos. This product offers good features, including labeling support through built-in AI models. TrainingData also supports DICOM, a widespread format in the medical domain.

In 2016 Radiology Informatics Laboratory Contour (RIL-Contour) was published [32]. RIL-Contour is an annotation tool for medical image datasets. Deep Learning algorithms support it to label images for Deep Learning research.

The tool most similar to ours is Endometriosis Annotation Tool [33]. The software, developed by a group of developers and gynecologists, is a web-based annotation tool for endoscopy videos. In addition to the classic functions such as video controls, screenshots, or manual labeling of the images, the option of selecting between different endometriosis types is also offered here.

Nevertheless, most of these medical annotation tools are not suitable for our comparison as they only work with images or are too specialized. The most suitable would be Endometriosis Annotation Tool, but the application is focused on specific annotations for surgery and those do not allow the creation of bounding box annotations which are crucial for our gastroenterological annotations. Therefore, we choose a common, well-known state-of-the-art tool CVAT, for our comparison.

## Results

This section presents the results of our introduced tool FastCAT and compares it to the well-known state-of-the-art annotation tool CVAT. We start by introducing our data acquisition and experimental setup. We show our results of the non-expert annotators, which suggests that our tool outperforms the state-of-the-art tool CVAT. We further show how the semi-automated AI annotation affects the annotation speed. Finally, we show our results of the expert annotator, which underline the time advantage using our tool.

### Data acquisition and experimental setup

For our evaluation, we used two data sets: The GIANA data set and our data set created at a German clinic called “University Hospital Würzburg”^2^. The GIANA dataset is openly accessible^3^ [34]. It is the first polyp dataset published, which includes videos. Former open-source datasets like CVC clinic database [35] or ETIS-LaribPolypDB [36] only provide single images. The GIANA dataset consists of 18 annotated polyp sequences. It is a standard dataset that has been used before for model benchmarking in different publications [37–39]. Therefore, we can reliably use it for evaluating the quality of our results. On average, the data set has 714 frames per video. According to their references, all annotations are done by expert gastroenterologists. We randomly selected two videos from the 18 available ones in GIANA for our evaluation, which turned out to be videos number 8 and 16.

Our data set is composed of an additional 8 videos. These videos include full colonoscopies and therefore have to be filtered first. For the filtering process, we used the method introduced in this paper. Furthermore, we contacted an expert gastroenterologist from the University Hospital Würzburg for the expert annotation. Since the expert annotation time of gastroenterologists is very costly and difficult to obtain, we could only manage to receive the work of two experts. In a second process, the expert annotators select the part of the video, including polyps, as explained in section Methods. However, since this annotation process is not yet completed, we can only evaluate the improvement in annotation speed and not the annotation quality with our dataset.

For the prospective study, all participants receive ten videos for polyp annotation. The videos are randomly selected and then given to the participants. For our preliminary evaluation, ten non-expert annotators are instructed to use our annotation tool and the state-of-the-art annotation tool CVAT. Finally, all non-expert annotators receive our software FastCAT and a java tool for measuring the time. The expert annotator starts with annotation, as explained in “Methods”. He annotates Paris classification [40], the size of the polyp, and its location. Additionally, the expert annotates the start and end frame of the polyp and one box for the non-expert annotators. Afterwards, the AI calculates predictions on these frames. The results of the AI are given to the non-expert annotators, who then only correct the predicted boxes. The non-expert annotators in this experiment are students from computer science, medical assistance, and medical secretary. All non-expert annotators are instructed to annotate the polyp frames as fast and as accurately as they can.

**Table 2:** Comparison of FastCAT and CVAT by video. This table shows our comparison of the well-known CVAT annotation tool to our new annotation tool FastCAT in terms of annotation speed. Videos 1 and 2 are open source and annotated. Videos 3–10 are from the University Hospital Würzburg

	Speed (SPF)		Total time (min)		Video information		
	CVAT	FastCat	CVAT	FastCat	Frames	Polyps	Framesize
Video 1	3.79	1.75	23.43	10.82	371	1	384x288
Video 2	4.39	2.49	32.85	18.63	449	1	384x288
Video 3	2.82	1.42	60.11	30.27	1279	1	898x720
Video 4	4.09	2.00	56.85	27.80	834	1	898x720
Video 5	4.57	2.39	53.24	27.84	699	2	898x720
Video 6	1.66	0.61	18.01	6.62	651	1	898x720
Video 7	1.70	0.64	11.22	4.22	396	1	898x720
Video 8	1.55	0.76	34.13	16.73	1321	2	898x720
Video 9	1.87	0.88	34.91	16.43	1120	1	898x720
Video 10	2.74	0.92	77.68	26.08	1701	4	898x720
Mean	2.92	1.39	40.24	18.54	882	1.5	795x633

**Table 3:** Comparison of FastCAT and CVAT by user. This table shows our comparison of the well-known CVAT annotation tool to our new annotation tool FastCAT in terms of quality of annotation and annotation speed. The quality metric is the F1-score. We count a TP if the drawn box matches the ground truth box more than 70 %

	Quality (%)		Speed (SPF)		Total time (min)		Medical Experience
	CVAT	FastCat	CVAT	FastCat	CVAT	FastCat	
User 1	99.30	99.50	7.33	3.71	48.78	25.30	Low
User 2	98.85	98.90	3.47	1.88	23.38	13.70	Low
User 3	97.97	98.51	4.59	1.53	31.28	11.17	Low
User 4	98.93	99.75	5.12	2.57	33.96	16.53	Middle
User 5	98.53	98.83	5.41	2.49	37.00	18.10	Middle
User 6	98.52	99.23	4.04	3.24	27.90	24.95	Low
User 7	99.45	99.30	5.20	2.70	35.01	21.28	Middle
User 8	99.35	99.08	5.25	2.86	33.90	19.57	Low
User 9	99.12	98.54	4.12	2.25	27.12	14.99	Low
User 10	98.93	99.48	5.63	2.76	37.53	19.89	Low
Mean	98.98	99.03	5.79	2.93	33.59	18.55	Low

### Results of the non-expert annotators

We evaluated the tool with 10 different gastroenterological videos containing full colonoscopies. The results are shown in Table 2 and in Table 3. As mentioned previously, we only evaluate the quality of the annotation in two videos from the openly accessible GIANA dataset. The accuracy of the annotations is thereby calculated by comparing the ground truth box of the already annotated open-source GIANA dataset with our newly created annotations. The quality evaluation is done via the F1-score. The F1-score describes the harmonic mean of precision and recall as show in following equations:

$$\begin{aligned}&\text {Precision} = \frac{{\text {TP}}}{{\text {TP}}+{\text {FP}}} \;\;\;\; \text {Recall} = \frac{{\text {TP}}}{{\text {TP}}+{\text {FN}}}\\&F_1 =\frac{2*\text {Precision}*\text {Recall}}{\text {Precision}+\text {Recall}} = \frac{2*{\text {TP}}}{2*{\text {TP}}+{\text {FP}}+{\text {FN}}}. \end{aligned}$$

We count an annotation as true positive (TP) if the boxes of our annotators and the boxes from the GIANA dataset have an overlap of at least 70%. Our experiments showed high variability between individual experts. We, therefore, concluded that a higher overlap is not attainable. Hence, to ensure reasonable accuracy, we choose an overlap of 70% which has been used in previous studies [41–43]. To determine annotation speed, we first measure the speed of the non-expert annotators in seconds per frame (SPF). On average, our annotators take 2.93 s for annotating one image while maintaining a slight advantage in annotation quality. Overall, our semi-automated tool’s annotation speed is almost 2x faster than the CVAT annotation tool, with 5.79 s per image. In addition, we evaluate the average time non-expert annotators spend annotating an entire video. The average video takes 18.55 min to annotate. In comparison, using the CVAT tool takes 40.24 min on average per video. Due to some faulty prediction results of the AI, the annotators sometimes delete boxes and draw new boxes as some polyps may be hard to find for the AI. This leads to higher annotation time in the case where polyps are mispredicted. Nevertheless, our tool is self-learning, and increasing amounts of high-quality annotations improve the prediction quality of the AI. This, in turn, speeds up the annotation process further. We elaborate on this in detail in the following subsection. To include more information concerning the video data, we include the number of frames per video, the number of polyps per video, and each video’s frame size. The videos provided by our clinic (Videos 3-10) have a higher resolution and a higher frame rate than videos gathered from different institutes. Overall the quality evaluation results show that almost similar annotation results to those of gastroenterology experts are achieved. For speed, our tool outperforms the CVAT tool in any video. In two videos, our tool is more than twice as fast as the CVAT tool.

#### Learning process of the non-expert annotators

**Fig. 1:**
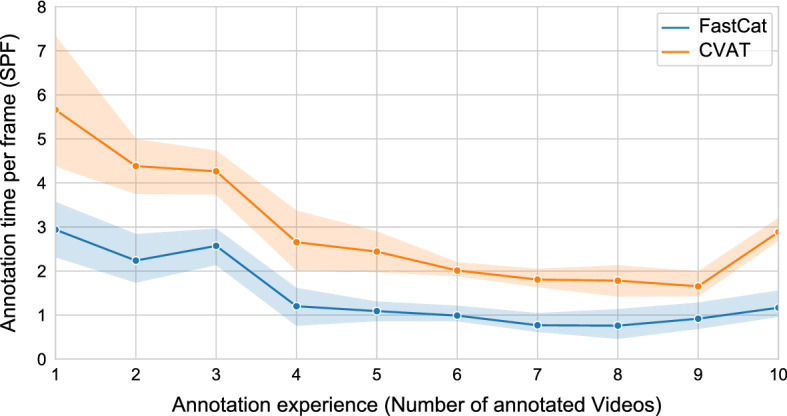
Learning process of the non-expert annotators. The figure shows the speed of the annotator in seconds per frame (SPF) over the annotation experience measured by the total number of annotated videos by that point for both our tool and CVAT

Figure 1 shows the learning process of the non-expert annotators, in blue using our tool and in orange using CVAT. The figure shows that the annotation of the first videos takes longer than annotating the subsequent ones since the non-expert annotator has to get to know the software and needs to adjust the software to his preferences. Therefore, annotation speed using both tools improves by further usage, and both tools feature a similar learning curve. However, this learning process slows down after the annotation of about 4 to 5 videos. After this amount of videos, annotators are well accustomed to the software and can competently use most features. In addition, Fig. 1 shows that this learning process is faster using our tool in comparison to the CVAT tool. This may be due to the information provided before use, the calculation we built directly into the software, and our user-friendly environment. Besides all, the CVAT software also shows excellent progress in learning worth mentioning. We can even see annotators who use any of the two tools more frequently further improve their annotation speed up to 9 videos. However, after 8 to 9 videos, the annotation speed decreases. This may be due to two repetitions of the same process that may bore the non-expert annotator and, therefore, decrease annotation speed. Our data show that this effect is more prominent for CVAT than for our tool.

#### Impact of polyp pre-annotations

**Fig. 2:**
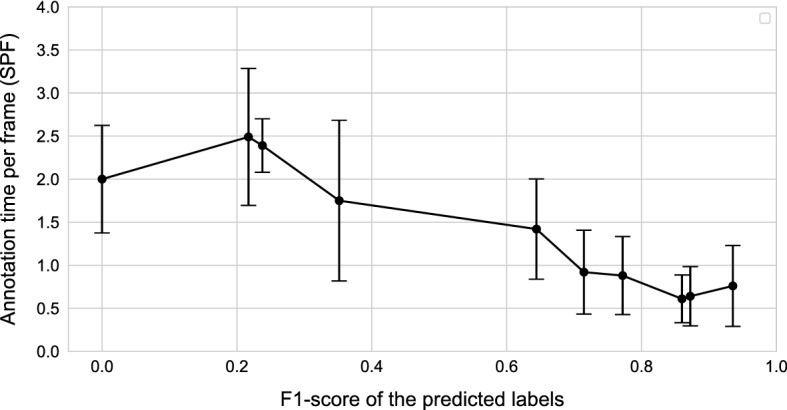
Effect of AI performance on annotation speed. Plotted are the speed of the annotators in seconds per frame over the AI performance given by its F1-score on a video-by-video basis, where the AI used for prediction is the same for each video. Every point is computed as the average over all annotators

To further analyze the improvements in our framework, we investigate the impact of polyp detection on the annotation speed. We compare the final annotated videos with the predictions done during the investigated videos. For ten videos, we calculated the F1-score based on the analysis above. A higher F1-score implicates more detected polyps with less false positive detection. Then, we rank the videos according to their F1-score and display the annotation speed in seconds per frame (SPF), shown in Fig. 2. Overall, a high F1-score leads to a faster annotation speed. Nevertheless, as seen in Fig. 2 if the F1-score is low, the annotation speed at times is faster without any predictions, e.g., from 0.2 to 0.4. Furthermore, low F1-scores show a higher standard deviation in the labeling speed. This means that with a higher F1-score, the variance of the non-expert annotators’ labeling speed decreases and therefore the overall performance is increased. Furthermore, we emphasize that continuing the annotation process and retraining the system detection results will increase, and therefore, the annotation speed will increase.

### Results of the expert annotators

This subsection demonstrates the value of the tool for domain expert annotation. As domain experts are very costly, we only had two experts available for our study. Therefore, our evaluation between domain experts could not be done quantitatively. Nevertheless, we can qualitatively compare the amount of time the domain experts took to annotate our collected colonoscopies. This is shown in Table 4. On average, our gastroenterologists spend 1.24 min on a colonoscopy. Our final results show that we achieved qualitatively similar results to the GIANA dataset annotation. The expert annotators only take 0.5 to 1 minutes per video using our method, while taking at least 10-80 minutes per video using the CVAT software. Therefore, we can reduce the amount of time a domain expert has to spend on annotation by 96.79 % or by a factor of 20 on our data. This reduction is primarily due to expert and non-expert annotation structure, which reduces the expert’s effort tremendously.

**Table 4:** Comparison of CVAT and FastCAT. The tables show the reduction of annotation time of the domain experts. Tgca stands for the time gained compared to annotation with CVAT and is the reduction of workload in %. Video 1 and video 2 are not used for this analysis as the open-source data do not provide full colonoscopies, but just polyp sequences and therefore it is not possible to perform an appropriate comparison

	Total time (min)		Tgca (%)	Video information		
	FastCat	CVAT		Length (min)	Freezes	Polyps
Video 3	0.50	60.11	99.15	15.76	2	1
Video 4	0.67	56.85	98.82	17.70	6	1
Video 5	1.09	53.24	97.95	23.12	4	2
Video 6	0.77	18.01	95.72	6.30	2	1
Video 7	0.70	11.22	93.79	13.05	5	1
Video 8	1.78	34.13	94.76	27.67	13	2
Video 9	1.50	34.91	95.70	20.53	4	1
Video 10	2.92	77.68	96.24	24.36	15	4
Mean	1.24	43.26	96.52	18.56	6.38	1.62

## Discussion with limitations

By implementing a novel workflow consisting of both algorithmic and manual annotation steps, we developed a tool that significantly reduces the workload of expert annotators and improves overall annotation speed compared to existing tools. In this section, we highlight and discuss the impacts of our study, show the limitation of our presented work and propose new approaches to advance our study further.

### Key features and findings

Our results show that by pre-selecting relevant frames using a combination of our freeze-frame detection algorithm and further, low-demand expert annotations and by using AI predictions for bounding box suggestions, we significantly increase the annotation speed while maintaining and even increasing annotation accuracy (see Tables 2 and 3). It is important to note that this improvement is not due to more annotation experience with one tool over the other since the annotators used the tools in an alternating fashion with random video order. Figure 1 further stresses this fact by showing a similar learning curve for both tools, with our tool being shifted down to shorter annotation times. In both cases, the annotation experience (i.e., adjustment to the tool) increases up to around seven videos or 10,000 annotated frames. The annotation speed first saturates and then increases again, possibly due to a human exhaustion effect of doing the same task for an extended duration [44].

Additionally, we inspected the effect of the prediction performance on the annotation speed. As shown in Fig. 2, there is a clear trend towards faster annotation time with better AI performance. The annotator works faster if the suggested bounding boxes are already in the correct location or only need to be adjusted slightly by drag and drop. If the predictions are wrong, the annotator needs to move the boxes further, perhaps readjust the size more, or even delete boxes or create new ones. However, the AI improvement saturates at an F1-score of around 0.8, where better AI performance does not equate to faster annotation speed. Additionally, the range of error is much more significant for the worse performing videos, so this point warrants further inspection in future studies. Nevertheless, it is apparent here that an AI only needs to be good enough instead of perfect to improve annotation speed significantly.

Finally, the results in Table 3 suggest that medical experience does not affect either the annotation speed or performance. The frame detection algorithm combined with the expert frame annotations and our AI’s pre-detection provides enough feasibility for the non-experts to adjust the suggested annotations fast and accurately regardless of experience. However, it should be noted that the range of speeds across our non-expert annotators is more stable for middle experience annotators than low experience ones.

All in all, our tool significantly improves the annotation workflow, specifically in the domain of gastroenterology, where specialized tools are scarce. The annotation speed is more than doubled while keeping the same accuracy as other state-of-the-art tools and keeping the cost for expert annotators low.

### Limitations of the study

In this subsection, we will shortly discuss the limitations of our analysis and provide an outlook for future studies.

First of all, we did not consider the difficulty of the video when analyzing annotation time. Some videos contain more and harder to detect polyps and thus provide a bigger challenge for both the AI and the annotator. The effect of video difficulty directly correlates to the AI performance in Fig. 2, where the standard error for low-F1 videos is much higher compared to the better ones. Some annotators can efficiently deal with false predictions, while others have more difficulties with those. Additionally, the total annotation time was measured from beginning to end for a video. While the applet we provided for the annotators includes a pause button, minor deviations, like checking their phone, are not removed from our total time measured. These statistical deviations could be removed by dividing the videos into difficulty categories and analyzing each category separately. We need more data or more annotators, where small statistical outliers should be averaged out.

Additionally, with only three medical assistants and seven non-experts, we need further tests to see if medical experience significantly affects annotation time and quality. As discussed above, Table 3 suggests that medium experience annotators work more consistently, whereas low experience ones can be both faster and slower than the medical assistants. These findings can be examined further in future studies with more annotators from various backgrounds, especially those with high medical experience.

Finally, we only indirectly measured the effect of bounding box pre-detection, where our non-expert annotators had no pre-detection for CVAT and suggestions with our tool. Thus, the improvement in annotation speed could also be due to our tool simply being easier to use and having a better user interface (UI) than CVAT. For future analysis, we intend to have the non-expert annotators annotate videos twice, once with bounding box suggestions and once without. However, both times they will use our tool. This way, we will be able to analyze the effect of the pre-detection directly.

### Limitations of the tool and future improvements

While our freeze-frame detection algorithm is specific to the domain of gastroenterology, the specific method for detecting relevant frames can be exchanged for a function more suited to the annotators’ domain. Additionally, while we only utilized the tool for polyp detection, it can be easily extended to feature more than one pathology, like diverticulum or inflammation. Since frame-wide annotations are separate from bounding boxes, this can also be used for standard image classification tasks and pathologies that are hard to confine to a bounding box area.

Additionally, within the medical domain, we plan to implement a feature for automatically detecting gastroenterological tools. When the acting doctor detects a suspicious polyp or other, they often remove them during the examination. The tools will then be visible on screen and are an indicator of pathology. Hence, the tool detection can be used as an algorithm to detect relevant frames within the videos.The pre-detection algorithm itself is also not limited to our deep learning AI trained on polyps but can be exchanged easily for a AI more suited to the user’s task.

The algorithm used for tracking objects across several frames is currently limited by the implemented standard object trackers above. These trackers are standard tools that often lose the object and have much room for improvement. While we provide an option for resetting the trackers, we intend to implement state-of-the-art video detection algorithms in the future to fully utilize this feature [45, 46].

## Conclusion

In this paper, we introduce a framework for fast expert annotation, which reduces the working amount of the domain experts by a factor of 20 on our data while retaining very high annotation quality. We publish open-source software for annotation in the gastroenterological domain and beyond. This includes two views, one for expert annotation and one for non-expert annotation. We incorporate a semi-automated annotation process in the software, which reduces time spent on annotation and further enhances the annotation quality. Our results suggest that our tool enhances the medical especially endoscopic image and video annotation, tremendously. We not only reduce the time spend on annotation by the domain expert, but also the overall effort.

## Methods

**Fig. 3:**
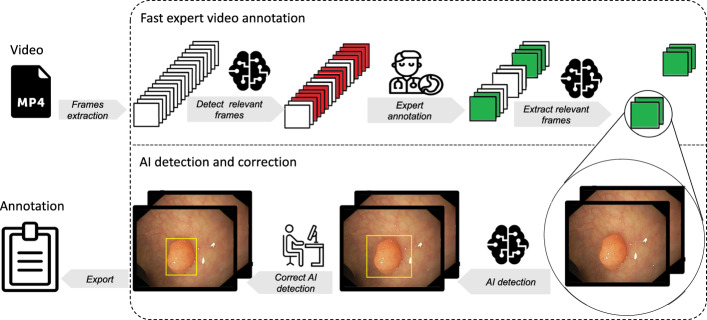
Annotation framework for fast domain expert labeling supported by an automated AI prelabeling

In this section, we explain our framework and software for fast semi-automated AI video annotation. The whole framework is illustrated in Fig. 3. The annotation process is split between at least two people. At first, an expert reviews the video and annotates a few video frames to verify the object’s annotations for the non-expert. In a second step, a non-expert has visual confirmation of the given object and can annotate all following and preceding frames with AI assistance. To annotate individual frames, all frames of the video must be extracted. Relevant scenes can be selected by saving individual frames. This prevents the expert from reviewing the entire video every single time. After the expert has finished, relevant frames will be selected and passed on to an AI model. This information allows the AI model to detect and mark the desired object on all following and preceding frames with an annotation. Therefore, the non-expert can adjust and modify the AI predictions and export the results, which can then be used to train the AI model.

### Input

To annotate individual video frames, the program must have access to all frames of the video. If annotated frames already exist, the program can recognize this; otherwise, it will extract all frames from the video and save them into a separate folder. Relevant frames can be annotated manually or inferred automatically. To mark the frames manually, frame numbers or timestamps are entered in the program. In the context of our polyp detection task, we created a script that detects when the recording freezes and marks these frames as relevant. A video freeze is caused by photos taken of suspicious tissue or polyps that are taken during the examination. The endoscope is stabilized mechanically if the examiner is pushing a button to take the photo. Therefore, these parts of the video are most relevant for the expert. This reduces the expert’s workload since he does not have to review the entire video, but can quickly jump to the relevant parts of the video. The extraction is done by using the OpenCV framework.

### Detect relevant frames

We denote all frames that assist the expert in finding critical parts of the video as *freeze frames*. Such frames can be detected automatically or entered manually by a frame number or timestamp. During a colonoscopic or gastroscopic examination, when the acting doctor detects a polyp (or similar), they freeze the video feed for a second and capture a photo of the polyp. Hence, for our task (annotation in gastroenterology), we automatically detect all positions in which a video shows the same frames for a short time, i.e., where the video is frozen for a few frames. Overall, within our implementation, we call such a position a “freeze frame”. The detailed explanation for detecting those freeze frames is shown in Algorithm 1.

In order to discover those freezes automatically, we extract all frames from the video using OpenCV [47]. OpenCV is one of the most famous computer science libraries for image processing. Afterwards, we compare each frame to its next frame. This is done by computing the difference in pixel values of both frames, converting it into the HSV color space, and calculating an average norm by using the saturation and value dimension of the HSV color model. A low average norm means that both frames are almost identical; hence a freeze could have happened. We save a batch of ten comparisons for a higher certainty and take an average of the ten last comparisons (similar to a moving average). If the average value falls below a certain threshold, we define the current frame as the start of a freeze. The end of a freezing phase is determined if the average value exceeds another defined threshold. This algorithm has high robustness and consistency as it rarely misses a freeze or creates a false detection.
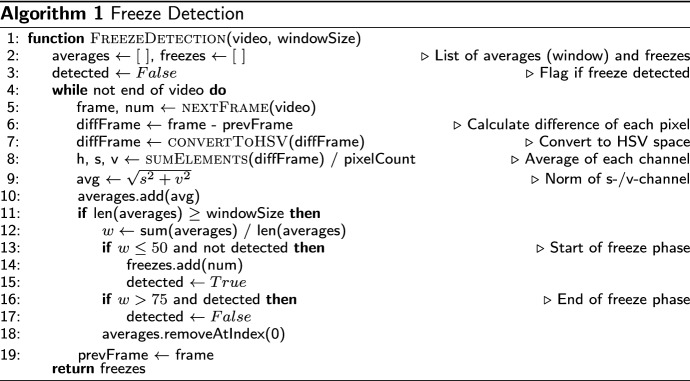


### Expert view

We refer to this part of the program *Video Review*, as the expert reviews the video to find polyps. For the expert to perform their task, they require the examination video, all individual video frames, and a set of relevant frame numbers, e.g., freeze frames. The video allows the expert to review the performed examination and get an overview of the presented situation to diagnose polyps correctly. All extracted video frames are necessary to be able to access and annotate individual frames. Lastly, a set of relevant frame numbers is given to the expert to jump to relevant video parts quickly. This led to a solution that provides the expert with two different viewpoints: (1) video player and (2) frame viewer. To enable fast and smooth transition between both viewpoints, it is possible to switch at any point in time from the current video time stamp *t* to the corresponding video frame *f* and vice versa. This is done by a simple calculation based on the frames per second (FPS) of the video and the current timestamp in milliseconds: $f = \frac{t\text {[ms]} \cdot \text {FPS[1/s]}}{1000}$.

It is possible to look at individual video frames within the frame viewer, assign classes to these frames, and annotate polyps within those frames. The class assignment is done through freeze frames, where each frame to which a class is assigned will be associated with a previously selected freeze frame. The second task, frame annotation, is independent of a class assignment and annotates the polyps within a frame with a bounding box that encloses the polyp. This primarily serves as an indication for non-experts to get visual information about the polyp that can be seen in the following/subsequent frames.

We use classes to mark frames if there is a polyp in the picture; we use these classes to mark relevant frames for the following annotation process by a non-expert. Two different approaches can be used to assign classes to frames. A range of frames is defined in the first approach by assigning start and end classes to two different frames. Consequentially, all frames in-between belong to the same class. The tool is also capable of assigning classes to each frame individually. The changes within video frames are small; therefore, many consecutive frames must be annotated with the same class. To make this process less time-consuming, the program allows the expert to go through a sequence of frames quickly and smoothly while classifying them by keeping a key pressed on the keyboard. However, mostly the assignment of start and end classes is faster and preferred.

Because all frames are mostly stored on an HDD/SSD, the loading latency is a performance bottleneck. We implemented a pre-loading queue that loads and stores the upcoming frames into the RAM to achieve fast loading times. This allows to display and assign frames with low latency. To prevent the queue from emptying rapidly, which causes high loading latency, we need to control the queue access times between two frames. Therefore, we use a capacity-dependent polynomial function to calculate a pausing time between frames: $\text {ms} = 50 \cdot ( 1 - \text {capacity} ) ^ {2.75}$. A full queue shortens the waiting time to 0 ms, while an empty queue leads to a 50-ms waiting time. This method combines fluent viewing and class assigning while providing enough time in the background to load new frames continuously.

Since the basic information about the presence of a polyp on an image is not sufficient for non-experts, and we want to ensure high-quality annotations, the expert has to annotate samples of all discovered polyps. This will provide visual information of the polyp to non-experts, allowing them to identify these polyps in all following and preceding frames correctly. Scenes in which polyps are difficult to identify due to perspective changes and other impairments should also be exemplary annotated by experts to provide as much information as possible to non-experts.

**Fig. 4:**
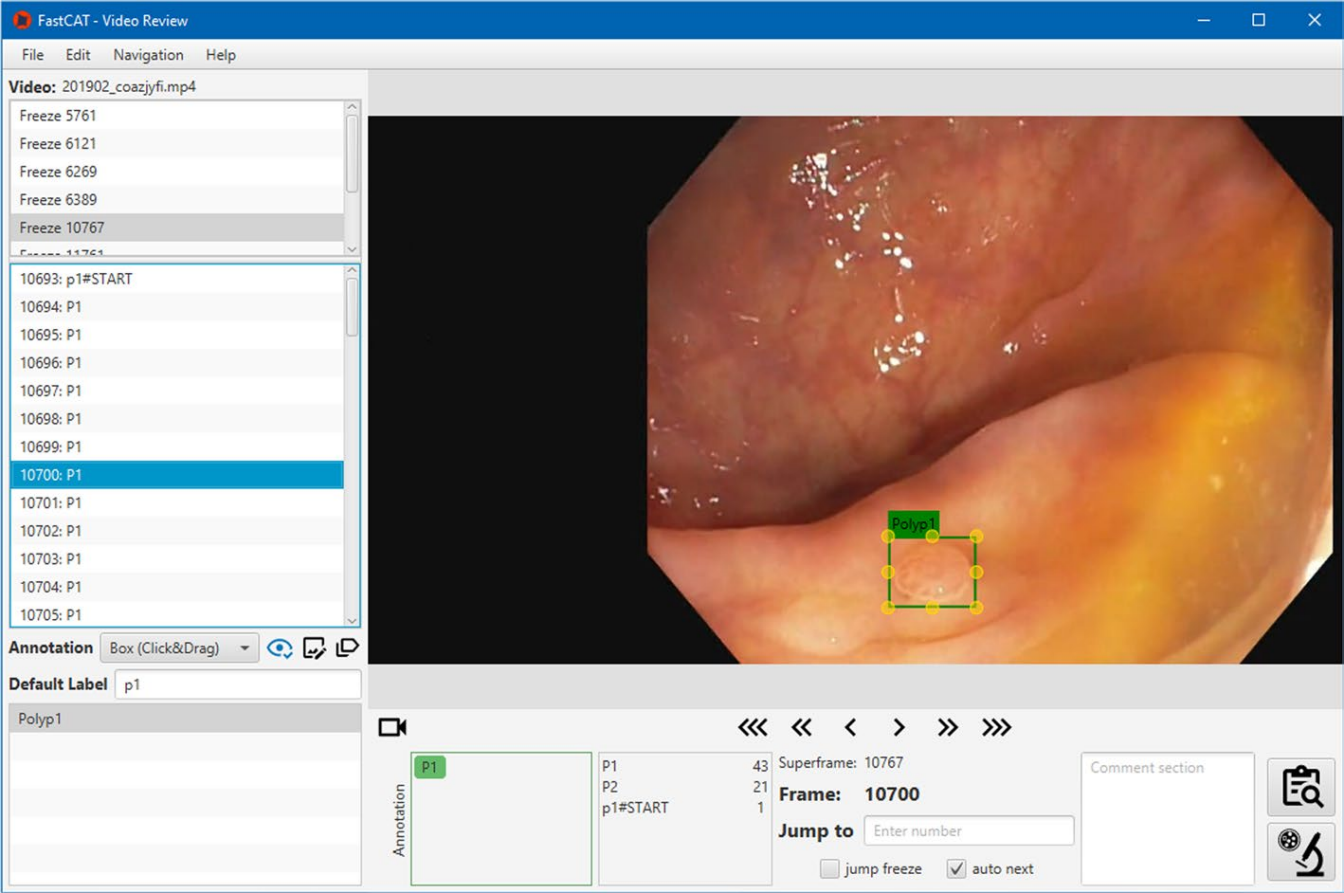
Video Review UI. The figure shows the list of freeze frames, the corresponding child frames, and annotations within the image on the right side. In the bottom part of the view, the user can insert comments, open reports, delete classes, and see all individual classes. The diseased tissue is delineated via bounding boxes

As we can see in Fig. 4 on the left side, the program lists all detected freeze frames. The list below shows all frames that belong to the selected freeze-frame and were annotated with specific classes, e.g., polyp type. Independent from the hierarchical structure above, we display all annotations that belong to the current frame in a list and on top of the image. In the lower part of the view, navigation controls skip a certain amount of frames or jump directly to a specific frame. The annotator can also leave a note to each frame if necessary or delete certain classes from the frame.

### Semi-automated polyp prelabeling

The prediction of polyps is made by an object detection model that was trained to detect polyps. The model we used is called EfficientDet [48]. EfficientDet is an object detection network that builds upon EfficientNet [49] and uses it as its backbone network. A feature extraction network is added on top of the backbone, which was named bidirectional feature pyramid network (BiFPN), and extracts the features of multiple layers. It is based on the idea of FPN and PANet [50] and combines multiple features of different sizes. This is called feature fusion and can be done by resizing or upsampling all feature resolutions to the same size and is combined by summing up. While previous methods did not consider the influence of a feature, BiFPN uses a weighted feature fusion that decides which features have the most influence. These features are then used for class and bounding box prediction. We adapted this network and trained it for polyp detection. The task of polyp detection is a combination of localizing and classifying an identified polyp. With this method, we aim for a fast AI-assisted annotation process for non-experts. Since every team has a different application, we distinguish between offline and online polyp prediction.

With an offline polyp prediction approach, we eliminate the need for high-end hardware for each user who uses AI assistance for fast annotation. The prediction is made by an external machine that is capable of running an AI model. With this approach, the extracted relevant frames are passed to this machine, generating a tool-specific JSON file that is then passed to the non-expert for further inspection.

As online polyp prediction, we define the performance of polyp detection locally on the machine of the annotator. Therefore, the machine on which our tool is executed must have the necessary hardware and software installed to run the detection AI. As there are different frameworks and deep learning networks, we need a unified interface to address all these different requirements. We decided to use Docker^4^ for this task. Docker uses isolated environments called containers. These containers only carry the necessary libraries and frameworks to execute a program. By creating special containers for each model, we can run a prediction independent of our tool and its environment. Containers are built from templates called images, which can be published and shared between users. Therefore, it is possible to create a repository of different models and prediction objectives. Because a container shuts down after every prediction, it must reload the model for the next prediction. To counteract this, we run a web server inside the container and communicate to the model via HTTP. This ensures that a model does not have to reload after every prediction and provides a universal and model-independent communication interface. With this setup, the user can trigger a single prediction or run a series of predictions in the background.

As we have already stated, we use HTTP for our communication. This gives room for a hybrid solution, allowing predictions on an external server while retaining the user’s control. This combines the advantages of the external and local approaches, where the user is not required to have expensive hardware, nor is it necessary to have a separate, time-consuming prediction step. The docker container is now running during the annotation process and AI is running in the container while using the program. Therefore, the diseased tissue delineating bounding box is directly drawn as an annotation on the image. This annotation can then be corrected or redrawn in the process.

### Non-expert annotation

**Fig. 5:**
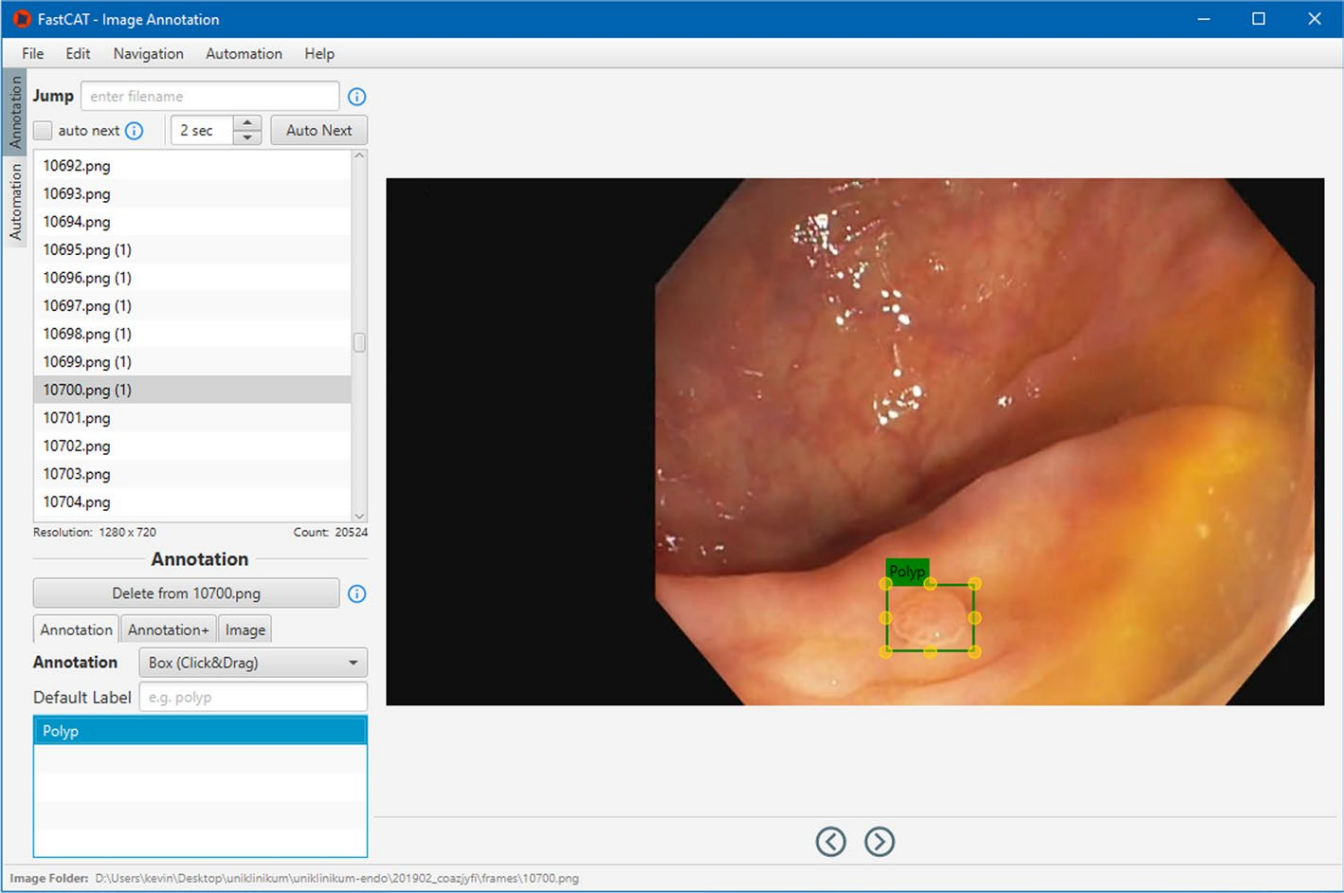
Image annotation UI. The figure shows a list of all available frames on the left with labeling functionality for a specific annotation and the whole image. The image to be annotated is displayed on the right. The diseased tissue is delineated via bounding boxes

With the help of AI, it is possible to annotate a large number of frames quickly and easily. The AI is predicting the annotations directly to the image. However, this method does not ensure the correctness of the predicted annotations. For this reason, these annotations must be checked and modified if necessary. Non-experts can check these predictions or create new annotations with the help of verified example annotations from the expert and the indication in which frame a polyp is visible. Besides, the AI-assisted support of our tool provides annotation duplication across several frames and object tracking functionality which speeds up the annotation process. Figure 5 illustrates the UI of the non-experts view.

As mentioned in section *Semi-automated polyp prelabeling* our tool supports the integration of AI detection. It can trigger a single prediction or make predictions on the following frames in the background. This enables the user to immediately annotate the remaining frames without waiting for the external prediction process to finish.

Another helpful feature is the duplication of annotations. Sometimes, only subtle movements occur in polyp examination videos, causing a series of frames to only show minuscule changes. This feature allows the non-expert to use the bounding boxes of the previous frame and only make minor adjustments while navigating through the frames. Re-positioning an existing bounding box requires less time than creating an entirely new box with a click and drag motion.

Our last feature uses object tracking to track polyps throughout consecutive frames. This avoids the manual creation of bounding boxes for each video frame, especially in sequences where an object’s visual and spatial transition between two frames is non-disruptive. For this task, we used trackers available in the OpenCV framework. Within the intestine, special conditions are usually present. First, the nature of colonoscopies leads to unsteady camera movement. Second, the color of polyps is often similar to the surrounding intestinal wall, which can make them hard to recognize. This can compromise the performance of the tracker and deteriorate polyp tracking. Given the fact that the annotation process requires a user to operate the tool and, therefore, the tracker does not need to track polyps fully automatically, we added two options to reset the tracker. This is described in more detail in the next section.

### Object trackers

As described in section *Non-expert annotation* our tool has object tracking functionality. It assists in tracking an object across multiple frames. For our tool, we implement six of the available trackers in the OpenCV framework [47]. In the following, we give a short description of the available trackers:

- *Boosting.* It is using an online version of AdaBoost to train the classifier. Therefore, the tracking is viewed as a binary classification problem, and negative samples of the same size are extracted from the surrounding background. It can update features of the classifier during tracking to adjust to appearance changes [51].
- *MIL.* Multiple Instance Learning uses a similar approach as Boosting and extracts positive samples from the immediate neighborhood of the object. The set of samples is put into a bag. A bag is positive when it contains at least one positive example, and the learning algorithm has to the inference which is the correct sample within a positive bag [52].
- *KCF.* Kernelized Correlation Filter uses the same basic idea as MIL, but instead of sampling a handful of random samples, it trains a classifier with all samples. It exploits the mathematical properties of circulant matrices to make tracking faster and better [53].
- *CSRT* CSRT uses discriminative correlation filters (CDF) with channel and spatial reliability concepts. The correlation filter finds similarities between the two frames. The spatial reliability map restricts the filter to suitable parts of the image. Scores estimate the channel reliability to weight features [54]. In addition, it is worth mentioning that rapid movements are not handled well by trackers that use CDF [55].
- *Median flow.* Median flow tracks points of the object forward and backward in time. Thereby, two trajectories are measured, and an error between both trajectories is estimated. By filtering out high error points, the algorithm tracks the object with all remaining points [56], It is best applicable for smooth and predictable movements [57].
- *MOSSE.* Minimum Output Sum of Squared Error is an adaptive correlation filter robust to light variation, scale, post, and deformations. It applies a correlation filter to detect the object in new frames. It works only with grayscale images, and colored images will be converted internally [58].
- *TLD.* TLD decomposes a long-term tracking task into tracking, learning, and detection. The tracker is responsible for tracking the object across the frames. The detector finds the object within a frame and corrects the tracker if necessary, and the learning part of the algorithm estimates the error of the detector and adjusts it accordingly [59].

An object tracker is designed to follow an object over a sequence of frames by locating its position in every frame. Each tracker uses different strategies and methods to perform its task. Therefore, trackers have to be switched and tested when tracking different pathologies. It can collect information such as orientation, area, or the shape of an object. However, also many potential distractions can occur during tracking that can make it hard to track the object. Distraction causes are, e.g., noisy images, unpredictable motion, changes in illumination, or complex shapes. As a result, the performance of different trackers can vary between different domains and datasets. For this reason, our tool allows the user to choose the best tracker for their task and dataset. Because trackers are primarily designed to track objects across many frames automatically, the tracker may generate less accurate bounding boxes over time or entirely lose track of the object. Since the tracking conditions for polyp detection are complex and our tool uses a semi-automated solution, we implemented two additional options for the annotation task.

By default, the tracker is initialized by placing a bounding box around an object that should be tracked. Consequently, the tracker will find the object on one consecutive frame and place a bounding box around it. We found that the tracker loses track of the initialized polyp with a high number of consecutive frames. Therefore, we implemented options to reinitialize the tracker automatically. The first option reinitializes the tracker after every frame, giving the tracker the latest visual information of the polyp. The second option only initializes the tracker if the user changed the bounding box size. Both options ensure that the tracker has the latest visual information of the polyp since the user corrects misaligned bounding boxes.

### Output and conversion

We use JSON as our standard data format. The JSON prepared by the expert stores detected freeze frames with all corresponding frames that contain at least one class. Additionally, annotated frames are stored in the same file but independently from the class assignments. The resulting JSON from the expert annotation process serves as an intermediate output for further annotations. All annotations that are done automatically are annotated so they can be distinguished from the annotations done manually.

The non-expert produces the final output with all video annotations. This file contains a list of all frames with at least one annotation. The tool produces a JSON with a structure designated to fit our needs. However, since different models require different data formats, we created a *python* script that converts our format into a delimiter-separated values (DSV) file format. Via a configuration file, the user can adjust the DSV file to its need, e.g., convert it into YOLO format. It is also possible to convert the DSV file back to our format. This enables seamless integration of different formats. In the future, further predefined formats can be added.

## Data Availability

The first dataset used for the analysis of this article is available in the GIANA challenge repository (https://endovissub2017-giana.grand-challenge.org/). The second dataset used during the analysis is available from the corresponding author on reasonable request.

## Abbreviations

COCO: Common Objects in Context
CSV: Comma-separated values
JSON: JavaScript Object Notation
YOLO: You Only Look Once
XML: Extensible Markup Language
CVAT: Computer Vision Annotation Tool
TFRecord: Tensor Flow Record
FER: Frame extraction rate
HTML: HyperText Markup Language
DICOM: Digital Imaging and Communications in Medicine
AI: Artificial intelligence
GIANA: Gastrointestinal image analysis
SPF: Seconds per frame
Tgca: Time gained compared to annotation
UI: User interface
HSV: Hue, saturation, lightness
RAM: Random-access memory
HTTP: Hypertext Transfer Protocol
DSV: Delimiter-separated values

## Footnotes

1 https://github.com/fastcatai/fastcat.

2 https://www.ukw.de/en.

3 https://endovissub2017-giana.grand-challenge.org.

4 https://docker.com.
